# Chronic Intake of Energy Drinks and Their Sugar Free Substitution Similarly Promotes Metabolic Syndrome

**DOI:** 10.3390/nu13041202

**Published:** 2021-04-06

**Authors:** Liam T. Graneri, John C. L. Mamo, Zachary D’Alonzo, Virginie Lam, Ryusuke Takechi

**Affiliations:** 1 Curtin Health Innovation Research Institute, Curtin University, Perth, WA 6845, Australia; Liam.graneri@postgrad.curtin.edu.au (L.T.G.); j.mamo@curtin.edu.au (J.C.L.M.); Zachary.dalonzo@postgrad.curtin.edu.au (Z.D.); Virginie.Lam@curtin.edu.au (V.L.); 2 Curtin Medical School, Faculty of Health Sciences, Curtin University, Perth, WA 6845, Australia; 3 School of Population Health, Faculty of Health Sciences, Curtin University, Perth, WA 6845, Australia

**Keywords:** energy drinks, artificial sweetener, metabolic syndrome, cholesterol, triglycerides, blood glucose, insulin, body fat, brown adipose tissue

## Abstract

Energy drinks containing significant quantities of caffeine, taurine and sugar are increasingly consumed, particularly by adolescents and young adults. The putative effects of chronic ingestion of either standard energy drink, Mother^TM^ (ED), or its sugar-free formulation (sfED) on metabolic syndrome were determined in wild-type C57BL/6J mice, in comparison to a soft drink, Coca-Cola (SD), a Western-styled diet enriched in saturated fatty acids (SFA), and a combination of SFA + ED. Following 13 weeks of intervention, mice treated with ED were hyperglycaemic and hypertriglyceridaemic, indicating higher triglyceride glucose index, which was similar to the mice maintained on SD. Surprisingly, the mice maintained on sfED also showed signs of insulin resistance with hyperglycaemia, hypertriglyceridaemia, and greater triglyceride glucose index, comparable to the ED group mice. In addition, the ED mice had greater adiposity primarily due to the increase in white adipose tissue, although the body weight was comparable to the control mice receiving only water. The mice maintained on SFA diet exhibited significantly greater weight gain, body fat, cholesterol and insulin, whilst blood glucose and triglyceride concentrations remained comparable to the control mice. Collectively, these data suggest that the consumption of both standard and sugar-free forms of energy drinks induces metabolic syndrome, particularly insulin resistance.

## 1. Introduction

Metabolic syndrome describes morphometric, physiological, and metabolic aberrations that are associated with increased risk of atherosclerotic cardiovascular disease (CVD) and type-2 diabetes mellitus [1]. Features that characterise metabolic syndrome are obesity, dyslipidaemia, hyperglycaemia, hypertension, and endothelial dysfunction [2]. A positive energy balance through exaggerated dietary intake stimulates insulin secretion, commensurate with increased dietary derived substrates. This milieu promotes hepatic lipogenesis and secretion of lipoprotein triglyceride [2,3,4]. Insulin also stimulates expression of adipose tissue endothelial lipases, resulting in tissue specific liberation and cellular uptake of non-esterified fatty acids and deposition as adipose tissue triglyceride [5,6]. The accumulation of triglyceride within adipocytes promotes secretion of pro-inflammatory adipokines, which, if persistent, paradoxically suppresses cellular response to insulin [5,7]. If the latter occurs, blood glucose levels increase, creating a vicious cycle of insulin secretion, lipoprotein hypersecretion, adiposity and chronic inflammation [8].

Highly caffeinated, carbonated energy drinks were first marketed around Europe and Asia in the 1960s [9], and are particularly popular and increasingly consumed amongst adolescents and young adults [10]. The primary ingredients of popular energy drinks such as Red Bull^TM^, Mother^TM^, and Monster^TM^ are caffeine/guarana (160 mg/serve), taurine, (2000 mg/serve), B6/B12 vitamins (1.0 mg/0.5 µg/serve respectively), sugar (51 g/serve), or alternative artificial sweeteners (e.g., sucralose and acesulfame potassium (Ace-K)) [11]. Clearly, consumption of energy drinks is relevant to risk for and severity of metabolic syndrome, because of interactive potential effects via increased provision of energy from diet and subsequent adiposity [12], a consequence of changes in energy utilisation patterns [13,14,15,16], potential effects on blood-pressure and vascular tone [17,18,19,20,21,22], a consequence of potential changes in vascular architecture (e.g., advanced glycation end-products) [23,24,25], or exacerbation of endocrine disorders (hyperinsulinemia/insulin resistance) [26,27,28,29,30]. However, there is no current literature exploring potential chronic interactive effects of energy drinks on metabolic syndrome.

In this study, the putative effects of a standard full-complement energy drink, a sugar-free energy drink and a popular cola-rich soft drink (Coca-Cola^TM^) containing phosphates and sugar, but free of taurine, were studied in wild-type mice in the context of metabolic parameters. We also compared the effects of energy drinks with mice that were maintained on a Western-styled diet enriched in saturated fatty acids (SFA) as a positive control for metabolic syndrome.

## 2. Materials and Methods

### 2.1. Animals and Dietary Intervention

Wild-type C57BL/6J male mice were purchased at 5 weeks of age from the Animal Resources Centre (Murdoch, WA, Australia). After 1 week of acclimatisation, mice were randomly assigned to one of 6 study groups (*n* = 10 per group), each given a unique dietary intervention. The control group was given only water with standard maintenance chow (AIN-93M, Glen Forrest, Specialty Feeds, WA, Australia). The drink groups were given either Mother^TM^ energy drink (ED), sugar-free Mother^TM^ (sfED), or Coca-Cola^TM^ carbonated soft drink (SD); all diluted to 30% (*v*/*v*) in water. In order to replicate the higher level of social relevance in the context of energy drink consumption, the dose of each drink ingested was determined based on similar previous studies (50 mL/mouse/d) [31]. The SFA group received only water with a diet containing 40% total energy from cocoa butter (SF07-050, Specialty Feeds). The SFA + ED group mice were maintained on the SFA diet in combination with ED drinking solution. The major nutritional components of AIN-93M chow, SF07-050 diet, Mother^TM^, sugar-free Mother^TM^, and Coca-Cola^TM^ is presented in Table 1. The consumption of respective food and drink quantities were measured twice weekly.

All animals were kept in individually ventilated cages with 12h light/dark cycles, under controlled temperature (21 °C) and air pressure. All drinks and diets were available ad libitum. All procedures described in this study were approved by the Curtin Animal Ethics Committee (AEC Approval No. 2018-03).

### 2.2. In Vivo Body Fat Composition Analysis

After 13 weeks of dietary/drink intervention, five randomly selected mice from the study groups were subjected to body composition scan using Skyscan high resolution in vivo X-ray microtomography (Bruker, Billerica, MA, USA) at the Centre for Microscopy, Characterisation, and Analysis (Harry Perkins Institute North, Nedlands, WA, Australia). The mice were anesthetised with isoflurane gas and the entire thoracic and abdominal areas of the body were imaged by Skyscan with 40 kV with 383 µA intensity. Smoothing was set to 2 and ring artefact reduction was set to 6 in order to reduce the noise and artefacts. Beam hardening correction was set at 20% to optimise the overall contrast. Total body fat percentages, white adipose tissue (WAT) and brown adipose tissue (BAT) volumes were quantitated using 3-D rendering software CT-Analyser (Bruker), which measured the overall volume of total adipose tissue, WAT or BAT through specific tomography intensity grading of 3-D x-ray shadow projection reconstructions. Total adipose tissue, WAT and BAT were distinguished by their difference in contrast by the experienced technical staff.

### 2.3. Serum Sample Collection and Blood Glucose Measurements

Following the dietary/drink intervention and body composition scan, the mice were anesthetized with gaseous isoflurane and blood samples were collected via cardiac puncture. Whole blood was collected and was used to measure glucose by using a point-of-care Accu-Check Performa (Roche, Basel, Switzerland). Blood levels of glycated haemoglobin (HbA1c) were also analysed immediately after cardiac puncture by using DCA Vantage Analyzer (Siemens, Munich, Germany) as described previously [32]. Subsequently, blood samples were allowed to clot at room temperature for 30 min and were centrifuged for 10 min at 4000 rpm. Thereafter, the supernatant serum was aliquoted and stored at −80 °C for further analysis.

### 2.4. Serum Insulin Concentration Analysis

Insulin concentration in serum after 13 weeks of energy drink/dietary interventions was measured using Ultrasensitive Mouse Insulin ELISA kit (Mercodia, Winston Salem, NC, USA). The procedure conducted was as per the manufacturer’s instructions. Briefly, 25 µL of serum samples were incubated with 100 µL enzyme-conjugated antibody solution for 2 h at room temperature. The samples were subsequently incubated with 200 µL TMB substrate for 15 min, and then the reaction was stopped by adding 50 µL stop solution. Optical density was measured at 450 nm within 30 min after adding the stop solution (Ensight^TM^, PerkinElmer, Waltham, MA, USA). The results of the standards were used to construct a non-linear log curve, which was then used to interpolate the concentrations of the samples.

### 2.5. Determination of Serum Lipid Concentrations

Total serum cholesterol and triglyceride were measured using commercial colorimetric assay kits as per the manufacturer’s instructions (Randox, Crumlin, UK). Briefly, 2 µL of serum samples were incubated with 200 µL of assay reagent for 10 min at room temperature. Optical density was measured at 546 nm within 60 min after incubation with the assay reagent using Ensight^TM^. The results of the standards were used to construct linear regression lines, which were used to interpolate the lipid concentrations of the serum samples.

### 2.6. Flow Cytometry Beads Array for Inflammatory Cytokines in Serum

Serum concentrations of the inflammatory cytokines interleukin (IL)-4, IL-6, IL-10, and tumour necrosis factor (TNF)-α, were determined using a cytometric bead array from BD Biosciences (North Ryde, NSW, Australia). The procedure was conducted as per the manufacturer’s instruction, with some minor changes. Briefly, 10 µL of undiluted serum, 10 µL of capture beads mixture, and 10 µL of PE detection reagent, were mixed in a 96-well microplate and incubated in the dark for 2 h. After a wash, and centrifugation, the beads were resuspended in 200 µL wash buffer. Results were acquired using FACS Canto II (BD Biosciences, North Ryde, NSW, Australia) and the data were analysed by using FlowJo (BD Biosciences, North Ryde, NSW, Australia). The standards provided in the kit were used to construct a one-phase decay non-linear regression line, which was subsequently used to interpolate the sample results.

### 2.7. Statistical Analysis

The data was entered into GraphPad Prism (ver. 7.04 Graph pad Software, San Diego, CA, USA) and expressed as mean ± SEM. Data normality was tested, and statistical analysis was conducted using a one-way ANOVA for all results, with Fisher’s LSD post hoc multiple comparison test. Statistical significance was determined at *p* < 0.05.

## 3. Results

**Figure 1:**
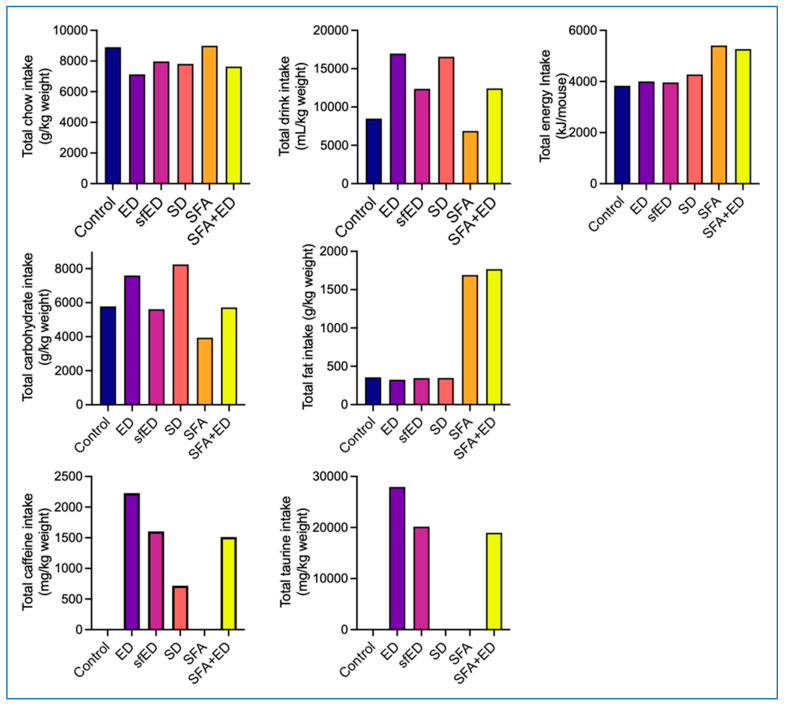
Chow, drink and nutrients consumption. The mice received no liquid intervention (control), Mother (ED), sugar-free Mother (sfED), Coca-Cola (SD), saturated fat enriched diet (SFA) or SFA with ED (SFA + ED) for 13 weeks. The chow and drink consumption was recorded twice weekly, and the total consumption over the 13-week experimental period is presented as per mouse. Statistical analyses were unable to be run because the data does not give errors.

The dietary and drink interventions were well tolerated and no adverse events were observed. Cumulative chow and drink intake per mouse over the 13 weeks of experimental period is presented in Figure 1. Based on the chow/drink consumption patterns, the cumulative energy, carbohydrate, fat, caffeine and taurine intake per mouse were determined. The intake was recorded twice weekly per cage where the mice were group housed. Thus, the data shown represent the estimated average per mouse and no error bars are given.

### 3.1. Energy Intake Patterns

The mice maintained on ED for 13 weeks consumed less chow compared to the control mice maintained on water. Instead, the ED mice consumed substantially more drink compared to the control mice, which led to a comparable total energy intake between the two groups. Similarly, the mice maintained on sfED consumed less chow and more drinks, which resulted in comparable energy intake to the control and ED mice. The mice maintained on SD for 13 weeks also consumed less chow, yet slightly greater than ED mice. The latter led to slightly increased total energy intake of SD mice, compared to the control, ED or sfED mice. The mice maintained on a diet enriched in SFA and water for 13 weeks showed comparable chow and drink consumption to the control mice with low-fat standard chow, leading to substantially higher total energy intake than the control, ED, sfED or SD group. The mice receiving a combination of ED and SFA diet consumed comparable feed and drink to sfED mice, which resulted in similar total energy intake to SFA group mice.

### 3.2. Macronutrient Consumption Profile

The consumption of ED or SD drink led to significantly increased total carbohydrate intake compared to the control mice (Figure 1). The consumption of sfED did not increase the carbohydrate intake, showing similar intake to control mice. As SFA chow contained less carbohydrate, the mice maintained on SFA showed lower carbohydrate intake compared to control mice. Such decrease in carbohydrate consumption of the SFA group was compensated by the ED intake in SFA + ED group mice, indicating comparable carbohydrate consumption to control mice, but lower than ED or SD mice. The consumption of ED, sfED or SD drink did not influence the total fat intake, showing comparable fat intake to the control mice (Figure 1). The mice maintained on SFA or SFA + ED showed significantly greater total intake of fat in comparison to the control mice receiving standard low-fat chow. The mice maintained on ED showed the highest intake of caffeine and taurine (Figure 1). Because the mice in the sfED and SFA + ED groups consumed a smaller amount of drinks compared to the ED group, their caffeine and taurine intake was also lower. The SD group mice consumed a similar amount of drink to the ED group, though the caffeine content in SD was significantly lower. Thus, the caffeine intake of SD mice was the lowest, compared to the mice in the ED, sfED and SFA + ED groups. The control and SFA group mice had no caffeine intake, and the control, SD and SFA mice had no taurine intake. Over the course of the experimental duration, there was no substantial changes in the consumption rate of diets or drinks across all experimental groups (Supplementary Figure S1).

### 3.3. SFA-Only and SFA + ED Mice Exhibited Markedly Greater Tissue Adiposity 

**Figure 2:**
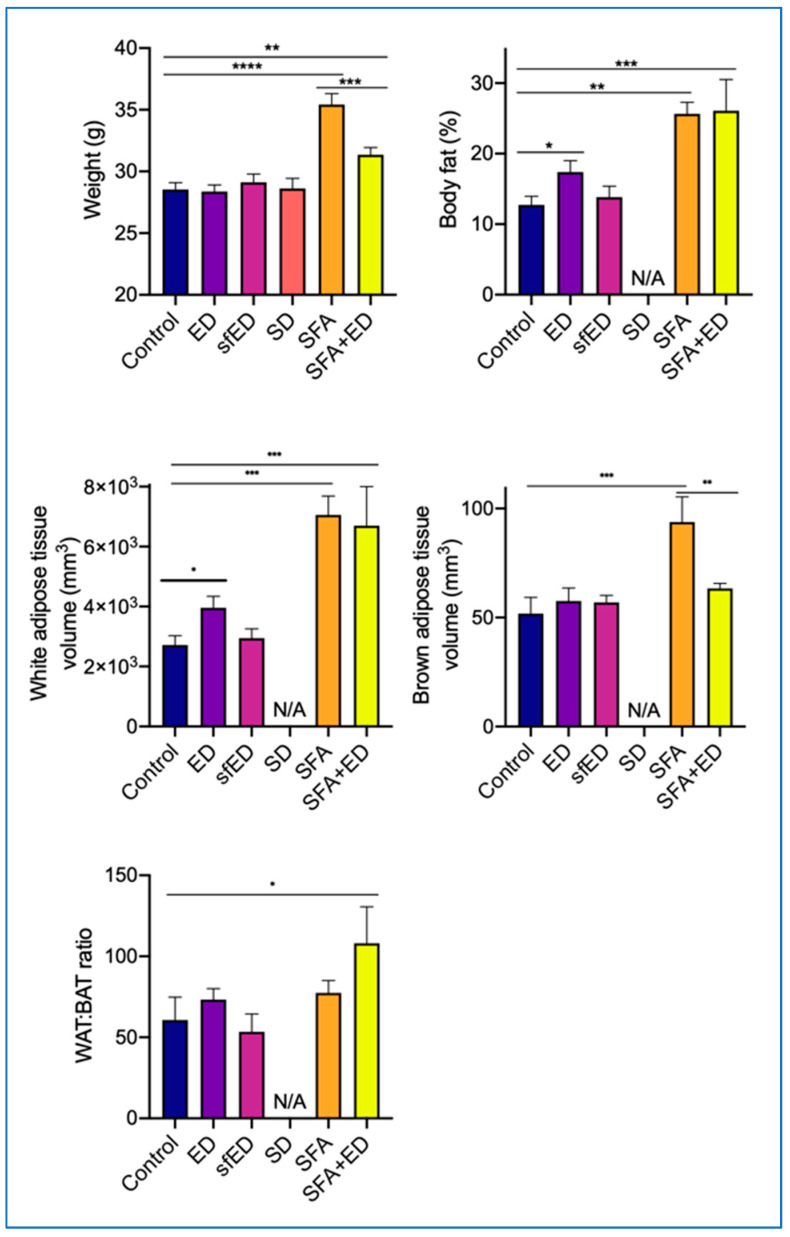
Weight gain and body fat. The mice received no liquid intervention (control), Mother (ED), sugar-free Mother (sfED), Coca-Cola (SD), saturated fat enriched diet (SFA) or SFA with ED (SFA + ED) for 13 weeks. The mice were weighed weekly. The body fat percentage, white adipose tissue (WAT) volume, brown adipose tissue (BAT) volume and WAT:BAT ratio were recorded from five randomly selected mice per group (except for SD group) by using Skyscan in vivo x-ray microtomography. Statistical significance was tested using a one-way ANOVA with Fisher’s LSD post hoc test (* *p* < 0.05, ** *p* < 0.005, *** *p* < 0.0005, **** *p* < 0.0001).

**Figure 3:**
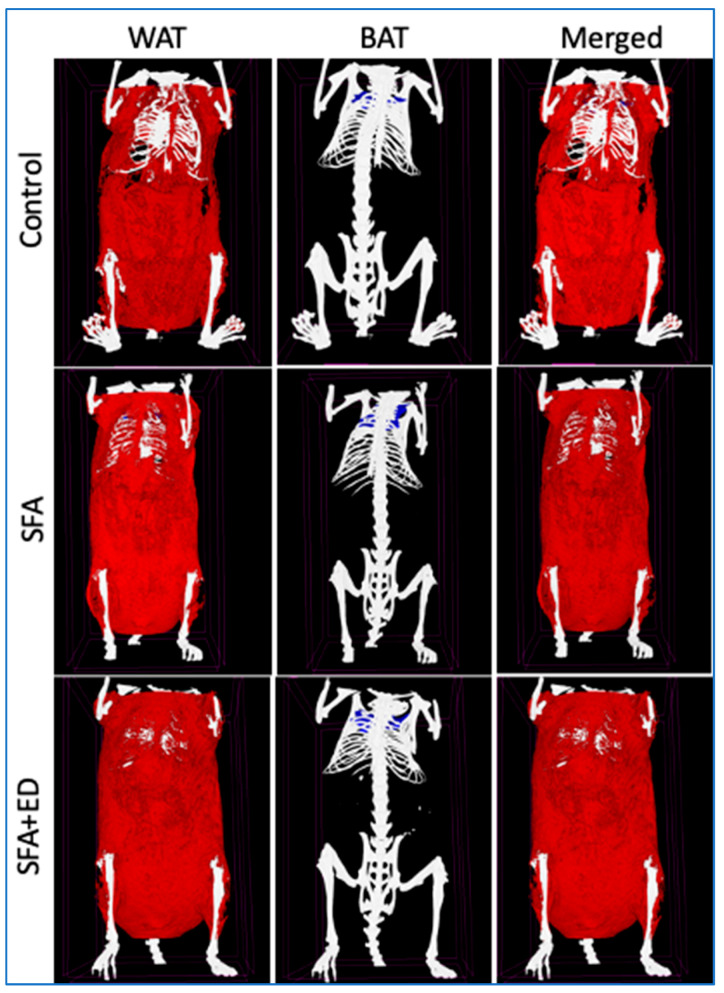
Representative Skyscan images of adipose tissue. Representative Skyscan x-ray microtomography images for the mice received no liquid intervention (control), saturated fat enriched diet (SFA) or SFA with ED (SFA + ED) for 13 weeks are presented. White adipose tissue (WAT) is shown in red while brown adipose tissue (BAT) is shown in blue.

The final weight of mice receiving ED intervention for 13 weeks was comparable to the control mice receiving water (Figure 2). The intervention with sfED also showed no significant effects on weight gain compared to the control mice. Interestingly, the mice maintained on ED showed significantly greater body fat percentage, compared to the control mice, whilst the body fat of sfED mice was comparable to the control (Figure 2). Further in vivo imaging analyses revealed that this increase in body fat induced by the ED intake was mainly due to the increase in WAT (Figure 2 and Figure 3). The weight gain of mice maintained on SD was similar to the control group. The mice fed with a diet enriched in SFA diet for 13 weeks had significantly greater weight gain as well as significantly increased body fat compared to the control mice receiving low-fat chow. Our data indicates that both WAT and BAT were significantly increased in the mice fed with SFA diet, showing a comparable WAT:BAT ratio with the control mice. In contrary, the mice maintained on SFA diet with a provision of ED drink also showed significantly higher weight compared to the control, but it was significantly less than the mice receiving SFA diet only. Nonetheless, the body fat percentage of SFA + ED mice was significantly higher than the control group mice and was comparable to the SFA mice. Furthermore, consistently with the mice maintained on ED only, the increased body fat in SFA + ED mice predominantly stemmed from the WAT increase, leading to significantly greater WAT:BAT ratio compared to the control mice.

### 3.4. ED, sfED, and SFA + ED Interventions Induced Indicies of Metabolic Syndrome

**Figure 4:**
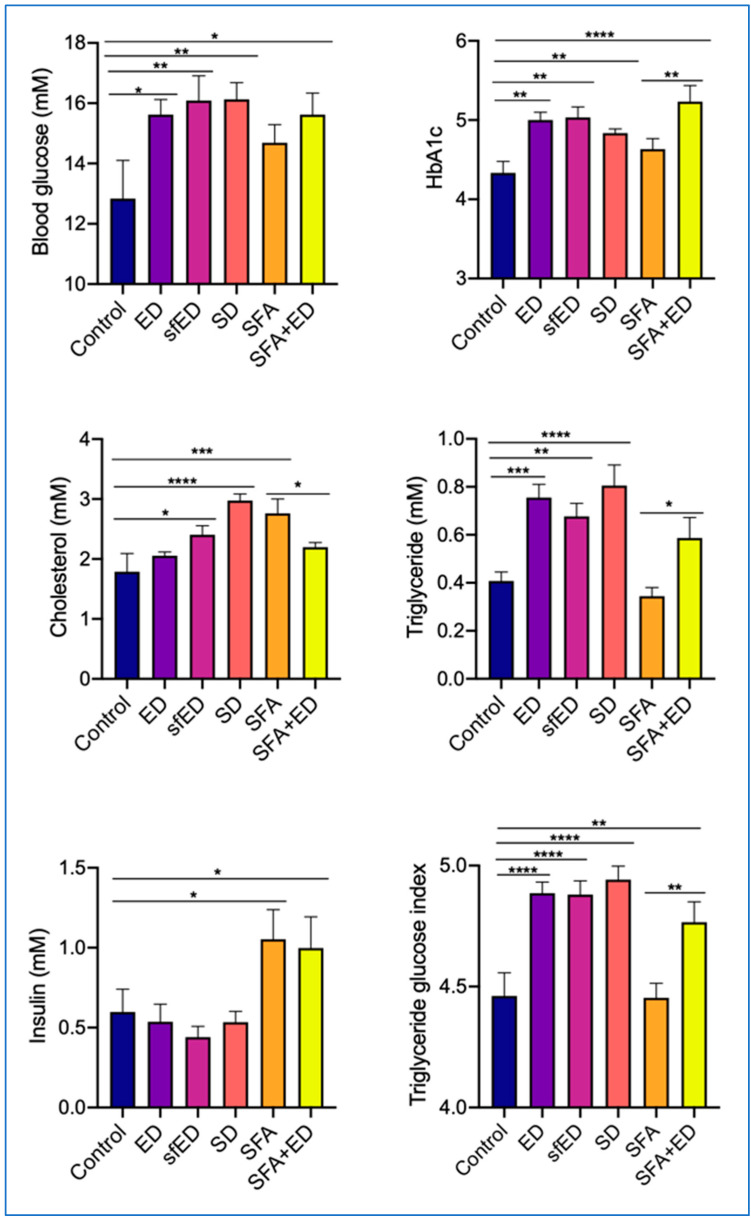
Blood glucose, lipids and insulin measurements. The mice received no liquid intervention (Control), Mother (ED), sugar-free Mother (sfED), Coca-Cola (SD), saturated fat enriched diet (SFA) or SFA with ED (SFA + ED) for 13 weeks. Blood glucose and HbA1c were measured at sacrifice with point-of-care device. Serum cholesterol and triglycerides were measured with colorimetric assay. Insulin was measured with a commercial ELISA kit. Triglyceride glucose index was calculated based on previous studies. Statistical analysis was performed using a one-way ANOVA with Fisher’s LSD post hoc analysis (*n* = 10; * *p* < 0.05, ** *p* < 0.005, *** *p* < 0.0005, **** *p* < 0.0001).

The mice receiving ED for 13 weeks had a significant increase in blood glucose, concomitantly with a significantly elevated HbA1c in comparison to the mice maintained on only water (control group) (Figure 4). Interestingly, the intake of sfED also resulted in a similar, significant elevation of blood glucose and HbA1c, compared to the control mice. The consumption of SD induced a significant elevation of blood glucose and HbA1c compared to the control mice, which was comparable to ED and sfED group mice. The ingestion of a SFA-enriched diet for 13 weeks moderately increased blood glucose and HbA1c, but it was not statistically significant. The combination of SFA and ED induced significantly increased blood glucose and HbA1c, compared to the mice maintained on a control low-fat chow or SFA diet, which was comparable to ED, sfED and SD group mice. The 13-week intervention with ED did not change the serum cholesterol concentration, whereas the serum triglyceride was almost double that of the control mice maintained on water (Figure 4). Interestingly, the mice maintained on sfED showed a significant increase in both serum cholesterol and triglyceride compared to the control. Similarly, the serum concentrations of cholesterol and triglyceride were significantly elevated in the mice receiving SD compared to the control mice. The ingestion of SFA-enriched diet significantly increased the serum cholesterol compared to the control mice with low-fat diet, whereas the serum triglyceride remained comparable. The mice maintained on SFA + ED showed comparable serum cholesterol to the control, which was significantly lower than the mice on SFA diet, whilst the serum triglyceride was significantly elevated compared to the SFA mice. Despite its significant effects on glucose, HbA1c and triglycerides, the ingestion of ED, sfED or SD did not increase serum insulin in comparison to the control mice receiving water (Figure 4). However, the mice receiving SFA diet for 13 weeks showed a significant increase in serum insulin compared to the control mice. Similarly, the mice maintained on SFA + ED combination had significantly higher serum insulin.

Triglyceride glucose index (TyG) is increasingly utilised as a marker of insulin resistance and diabetes. TyG was significantly elevated in the mice that were maintained on ED for 13 weeks, compared to the control mice receiving only water (Figure 4). Interestingly, the mice that were on sfED intervention for 13 weeks showed a similar significant raise in the TyG to ED group mice. The mice with SD also showed a significant TyG increase compared to the control mice. TyG of the mice fed with SFA diet was comparable to the control mice receiving standard low-fat chow, whilst the mice on a combination of SFA and ED intervention showed significantly elevated TyG compared to the control mice and SFA mice.

### 3.5. ED Elicits a Pronounced Inflammatory Cytokine Response 

**Figure 5:**
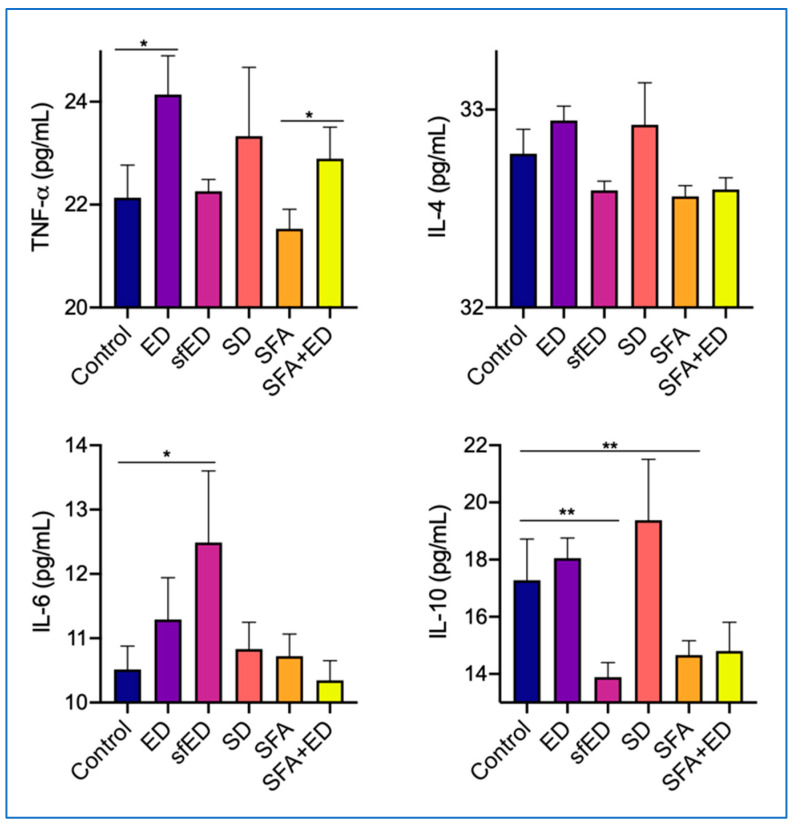
Serum cytokine concentrations. The mice received no liquid intervention (control), Mother (ED), sugar-free Mother (sfED), Coca-Cola (SD), saturated fat enriched diet (SFA) or SFA with ED (SFA + ED) for 13 weeks. Serum concentrations of inflammatory cytokines TNF-α, IL-4, IL-6 and IL-10 were measured using a commercial cytokine beads array kit. Interpolated values are presented as mean ± SEM for each cytokine. Statistical analysis was performed using a one-way ANOVA with Fisher’s LSD post hoc analysis (*n* = 10; * *p* < 0.05, ** *p* < 0.005).

The mice maintained on ED drink intervention for 13 weeks showed a significant increase in the serum concentration of TNF-α compared to the control mice receiving water, whilst the serum IL-4, IL-6 and IL-10 in ED mice were comparable to the control (Figure 5). In the mice receiving sfED, serum IL-6 was significantly elevated compared to the control group, whereas the serum IL-10 was significantly decreased by the ingestion of sfED. The serum concentrations of TNF-α and IL-4 in sfED mice were comparable to control mice. The ingestion of SD for 13 weeks did not significantly alter the serum concentrations of TNF-α, IL-4, IL-6 or IL-10. The mice maintained on SFA diet showed a significant reduction in IL-10 compared to the mice receiving low-fat control chow and water. The serum concentrations of TNF-α, IL-4 and IL-6 in SFA group mice were similar to the control mice. Similar to the ED group mice, the mice maintained on a combination of SFA diet and ED showed a significant increase in the serum TNF-α in comparison to the mice that were fed with SFA diet and water. The serum concentrations of IL-4 and IL-6 in the mice receiving a combination of SFA diet and ED were not significantly different from the control mice or SFA group. Similar to SFA-fed mice, serum IL-10 showed a moderate reducing trend in SFA + ED mice.

## 4. Discussion

The putative effects of chronic ingestion of either the standard energy drink, Mother^TM^ (ED) or its sugar-free formulation (sfED) on metabolic parameters were determined in wild-type C57BL/6J male mice in comparison to another popular soft drink, Coca-Cola^TM^ (SD), a Western-styled diet enriched in SFA, or a combination of SFA and ED (SFA + ED). The consumption of ED reduced the chow intake, leading to a similar energy intake between the control and ED/sfED groups. Similarly, the chow consumption of SFA group mice was reduced by ED intake, whilst the total energy intake of SFA + ED group mice remained substantially higher than the control or ED groups. In previous studies using rats, taurine was shown to reduce food intake [33], which is consistent with our observation. However, in our study, the intake of SD also suppressed the chow consumption where no taurine was included. The reduced food intake in SD group mice may be attributed to the caffeine intake. A clinical study demonstrated that a small amount of caffeine consumption (1 mg/kg) significantly reduced the food intake by 10% in healthy adult participants [34]. Another study also showed that the moderate consumption of coffee reduces the ad libitum meal intake in overweight/obese individuals [35]. Whilst the mechanisms whereby caffeine reduces food intake is not fully understood, a study using mouse models revealed that caffeine acts as an antagonist for the adenosine receptor A_1_R in the paraventricular nucleus of the hypothalamus and suppresses the appetite [36]. Hosoi et al. reported that caffeine reduces the food intake by attenuating leptin resistance in neurons by inhibiting endoplasmic reticulum stress [37]. Taken together, our data indicated that the consumption of highly or moderately caffeinated drinks irrespective of their taurine content may attenuate the appetite and consequently reduce the overall food intake.

A reduction of the chow consumption in ED, sfED and SD group mice led to a similar total energy intake to the control mice maintained on water. Thus, the weight gain amongst these groups was also comparable. However, the mice in the ED group showed significantly greater body fat percentage compared to the control group. In the SFA-fed mice, whilst the final weight was significantly less than SFA + ED mice compared to the SFA only mice, the body fat percentage of the two groups was comparable, which was significantly higher than the control mice. Interestingly, such an increase in body fat was observed only in the ED groups, but not in the sfED group, indicating that the increased body fat may stem from the greater sugar intake. Indeed, the mice in the ED group had greater carbohydrate intake compared to the mice in the sfED group, whilst their fat intake was comparable. Similarly, the SFA + ED group showed greater carbohydrate intake compared to the SFA group, while the fat intake was similar. Furthermore, our in vivo Skyscan imaging revealed that the consumption of ED results in the significant increase of WAT volume and WAT:BAT ratio, whereas the consumption of SFA diet increases both WAT and BAT volume. In contrast to WAT, BAT is widely reported as ‘good fat’, increasing thermogenesis and insulin sensitivity. A large body of literature report that caffeine enhances thermogenesis and energy expenditure, leading to fat loss [38]. Indeed, a study by Velickovic et al. demonstrated, both in vitro and in vivo, that an exposure to caffeine significantly increases adipocytes’ thermogenesis and induces browning-like structural changes in mitochondrial and lipid droplet content [39]. However, the majority of these studies used purified caffeine or caffeine in the form of coffee to explore its effects on adipocytes and did not consider the combination with sugar intake or the form of energy drinks. Our data suggests that the thermogenic effects of caffeine in the form of sugared energy drinks may be counteracted by its sugar content, resulting in a significant increase of WAT without evident weight gain.

Our results also showed a significant elevation of blood glucose in mice maintained on ED, sfED, SD and SFA + ED interventions, while the SFA diet only modestly increased the blood glucose. HbA1c was also significantly upregulated in ED, sfED, SD and SFA + ED mice. The indicated chronic blood glucose elevation as a result of ED and SD ingestion was accompanied by higher carbohydrate intake, and thus was a well-expected outcome [40]. In contrast, the observation of increased blood glucose and HbA1c in mice receiving sfED was particularly interesting because their carbohydrate intake was comparable to the control mice. The only difference between the standard Mother^TM^ and sugar-free Mother^TM^ is the content of sugar vs. artificial sweeteners, which are Ace-K and suclarose. A study reported that when a single serve of sugar is replaced by artificial sweeteners, it does not significantly influence the post-prandial and 24 h blood glucose homeostasis, insulin and energy intake [41]. However, a chronic long-term intake of artificial sweeteners were shown to have a positive association with fasting blood glucose levels [42]. A randomised double-blind controlled trial found that after 4 weeks of supplementation with sucralose, the subjects had a significantly decreased acute insulin response, decreased insulin sensitivity and increased release of glucagon-like peptide-1 [43]. Furthermore, a recent meta-analysis found that the consumption of caffeine increases the blood glucose and prolongs the period of high blood glucose [44]. Additionally, our study also found that a marker of insulin resistance, TyG, was significantly elevated in mice receiving ED, sfED, SD and SFA + ED interventions. These data collectively suggest that despite the lower carbohydrate intake, chronic consumption of sugar-free energy drinks may promote insulin resistance in a similar manner to the sugared energy drinks or soft drinks.

Serum insulin concentration is a significant factor driving metabolic syndrome. Paradoxical to the results indicating exaggerated insulin resistance in the ED group, in sfED and SD mice, there were no distinct changes observed in the serum insulin concentrations. Taurine has been demonstrated to ameliorate insulin resistance in animal studies in the context of diabetes [45]. However, in a long-term clinical study in pre-diabetic overweight men, daily supplementation with taurine had no beneficial effects on insulin secretion or sensitivity or blood lipid levels [46]. Moreover, a recent mouse study demonstrated that chronic taurine supplementation further enhanced high-fat induced insulin resistance and suppressed the insulin secretion by pancreatic β cells [47]. Furthermore, in an in vitro study using β cells, taurine was demonstrated to decrease the synthesis of insulin [48]. Despite this, the Coca-Cola^TM^ treatment group, which lacked a taurine component, had an analogous scarcity of hyperinsulinemia when compared to the control group, which implicates the involvement of caffeine. The intake of caffeine is reported to promote insulin resistance [44,49], whilst a consumption of decaffeinated coffee is shown to improve insulin sensitivity [50]. Consistently, maternal caffeine exposure was reported to impair the secretion of insulin by pancreatic β cells in offspring [51]. While some studies reported no impacts of caffeine on insulin sensitivity [52,53], a population study also found that the intake of caffeine significantly reduced the insulin secretion by ~27% [54]. These taurine and caffeine mediated pathways may explain the lowered serum insulin observed in the ED, sfED and SD supplemented mice, which may have further exacerbated blood glucose concentrations.

There is well established research encompassing all components of energy drinks that has consequences for lipid homeostasis. Elevated blood triglycerides is a characteristic of insulin resistance [55]. Concomitant with increased blood glucose and HbA1c, all groups of mice receiving ED or SD showed a significant elevation in serum triglyceride compared to the control mice receiving only water or SFA diet. It can therefore be speculated that insulin resistance, as suggested by the elevated blood glucose in ED and SD supplemented mice promoted the hepatic synthesis and secretion of VLDL, which thereafter resulted in the elevation of serum triglyceride. A national health survey on a German population found that caffeine intake had no association with total cholesterol or LDL levels; however, interestingly, a positive correlation between caffeine and triglycerides was reported [16], implicating the involvement of caffeine in the triglyceride response. Whilst the SD or SFA groups showed a significant increase in the serum concentrations of cholesterol, the mice receiving ED or sfED had attenuated levels of serum cholesterol. A potential reason for the lack of elevated cholesterol in energy drink-treated mice may be due to taurine’s suppressive effects on serum cholesterol [56]. An in vitro study using human hepatocytes found that taurine enhances the expression of CYP7A1 genes to promote intracellular cholesterol metabolism and reduces cholesterol secretion [57]. Collectively, our results suggest that the intake of energy drinks may significantly increase the serum levels of triglyceride and promote insulin resistance due to its sugar and caffeine content, while it may suppress the elevation of serum cholesterol.

Inflammation is regulated through both pro- and anti-inflammatory cytokines. The levels of TNF-α were markedly increased in the ED group and SFA + ED group mice compared to the control, whilst the SD mice showed a modest increase. As this was not significant in the sfED or SFA groups, which had comparable sugar intake to the control group, the effect of increased TNF-α was attributed to the net intake of the sugar components in ED or SD. This could be elucidated due to the AGE-RAGE interaction, which occurs in hyperglycaemic conditions and characteristically releases TNF-α [25]. While the anti-inflammatory cytokine IL-4 showed no net changes between all intervention groups compared to the control, a moderate decrease was observed in the sfED, SFA-only and SFA + ED mice. Similarly, the serum concentrations of anti-inflammatory IL-10 were markedly lower in sfED and SFA groups compared to the control group. Concomitant with the IL-4 results, the pro-inflammatory marker IL-6 was significantly increased in the sfED group mice. The mechanisms responsible for the reduced anti-inflammatory cytokines between sfED and SFA treated mice may substantially differ. Artificial sweeteners such as sucralose and Ace-K are reported to mediate the secretion of inflammatory cytokines by modulating its transcriptome expression [58]. Consistently with our findings, an in vitro study using whole blood culture assays showed significant increases in IL-6 and reduction in IL-10 by the addition of sweeteners including sucralose [59]. A significant reduction of anti-inflammatory IL-4 and IL-10 by artificial sweeteners has also been demonstrated in mouse lymphocytes [60]. In contrast, in SFA-induced inflammation, impaired insulin signalling is reported to be responsible for promoting inflammation [61]. A 15-week intervention with SFA-enriched diet in wild-type mice was shown to alter the liver expression of acetyl-CoA carboxylase-1, insulin receptor substrate-1, AMP-activated protein kinase and toll-like receptor-4, leading to significantly lower plasma levels of IL-10 [61]. These data suggest that the intake of ED, sfED, SD and SFA may differentially promote inflammation via alternate pathways.

## 5. Conclusions

In conclusion, our study indicates that the consumption of energy drinks increases the risk of metabolic syndrome, specifically increased white adipose tissue, inflammation and enhanced insulin resistance. Such detrimental effects of energy drinks appeared to be attributed to the exaggerated intake of taurine, caffeine and sugar. The ingestion of Western-styled diet enriched in SFA showed differential detrimental effects on metabolic health through distinct mechanisms. Our outcomes indicate that whilst sugar-free forms of the energy drink result in lower sugar intake, they may also promote substantial insulin resistance, dyslipidemia and inflammation in a similar manner to standard sugared energy drinks. These findings clearly indicate potential health concerns surrounding the increasing consumption of both standard and sugar-free energy drinks.

## Figures and Tables

**Table 1:** Nutrition composition table of energy drinks, soft drink, and chow.

Per 100 g or 100 mL		Control (AIN-93M)	ED (Mother^TM^)	sfED (Sugar-Free Mother^TM^)	SD (Coca-Cola^TM^)	SFA (SF07-050)	SFA + ED
Drinks	Energy (kJ)	0	57.3	5.7	54	0	57.3
	Carbohydrate, Total (g)	0	3.03	0.03	3.18	0	3.03
	Caffeine (mg)	0	9.57	9.57	2.91	0	9.57
	Taurine (mg)	0	120	120	0	0	120
	Vitamin B3 (mg)	0	0.54	0.54	0	0	0.54
	Vitamin B6 (mg)	0	0.06	0.06	0	0	0.06
	Vitamin B12 (ug)	0	0.15	0	0	0	0.15
	Vitamin B5 (mg)	0	0.198	0.198	0	0	0.198
Diet	Energy (kJ)	1510	0	0	0	1880	1880
	Carbohydrate, Total (g)	77.7	0	0	0	61.3	61.3
	Fat, Total (g)	4	0	0	0	20.3	20.3
	Fat, Saturated	0.3	0	0	0	13	13
	Vitamin B3 (mg)	3	0	0	0	3	3
	Vitamin B6 (mg)	0.7	0	0	0	0.7	0.7
	Vitamin B12 (ug)	10.3	0	0	0	10.3	10.3
	Vitamin B5 (mg)	1.65	0	0	0	1.65	1.65

## Data Availability

All data is presented and available in this manuscript.

## Supplementary Materials

The following are available online at https://www.mdpi.com/article/10.3390/nu13041202/s1, Figure S1: Diet and drink consumption over the course of the study.
