# Supplementary material for: Chronic Intake of Energy Drinks and Their Sugar Free Substitution Similarly Promotes Metabolic Syndrome
Source: Nutrients. 2021 Apr 6;13(4):1202. doi: 10.3390/nu13041202 (PMC8067378; doi:10.3390/nu13041202)

Chronic Intake of Energy Drinks and Their Sugar Free Substitution Similarly Promotes Metabolic Syndrome

Supplementary material:

Figure 1. Diet and drink consumption over the course of the study.

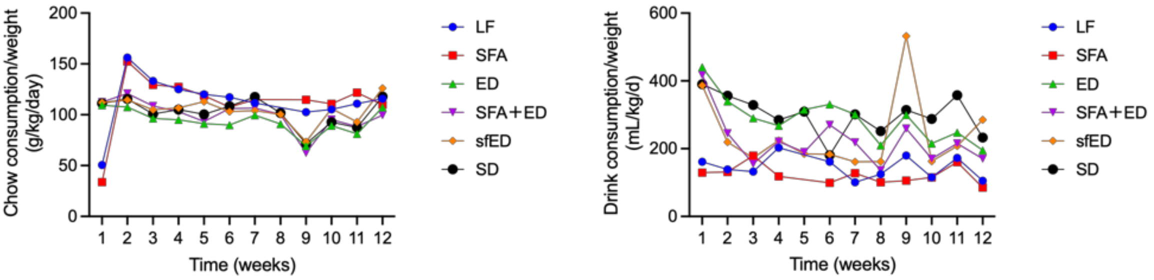

Supplement: Supplementary file 1 [file nutrients-13-01202-s001.pdf]
